# Assessment of Simplified Surveillance for Congenital Rubella Syndrome in Sudan, 2014–2017

**DOI:** 10.3390/vaccines12121447

**Published:** 2024-12-23

**Authors:** Omayma Abdalla, Nada Ahmed, Hanan Abdo El-Hag Mukhtar, Susan Reef, Jose Hagan, Gavin Grant

**Affiliations:** 1 Expanded Program for Immunization, Sudan Ministry of Health, Khartoum P.O. Box 303, Sudan; omeimah6@gmail.com (O.A.); nadajafar@gmail.com (N.A.); 2 Sudan Country Office, World Health Organization, Khartoum P.O. Box 303, Sudan; abdoh@who.int; 3 Global Immunization Division, United States Centers for Disease Control and Prevention, Atlanta, GA 30329, USA; susanreef4@gmail.com (S.R.);

**Keywords:** surveillance, congenital rubella syndrome (CRS), rubella vaccine introduction, rubella surveillance, congenital birth defects, surveillance trends

## Abstract

Background/Objectives: Congenital rubella syndrome (CRS) is a constellation of serious multi-organ birth defects following rubella virus infection during early pregnancy. Countries in which rubella vaccination has not yet been introduced can have a high burden of this disease. Data on CRS burden and epidemiology are needed to guide the introduction of a rubella vaccine and monitor progress for rubella elimination, but the multi-system nature of CRS manifestations and required specialized testing creates a challenge for conducting CRS surveillance in developing settings such as Sudan. To enhance data quality, we designed and tested a simplified approach for CRS surveillance in Sudan. Methods: Seven CRS surveillance sentinel sites were set up at general pediatric, eye, and cardiology hospitals in Sudan, using standard definitions for reporting and classifying infants with CRS clinical manifestations. Between 2014 and 2017, we evaluated the system using WHO CRS surveillance monitoring indicators, comparing simplified approaches against a comprehensive one. The simplified approaches included (1) an ophthalmic-focused approach; (2) a heart-focused approach; and (3) a cataract-only approach. Results: Surveillance identified 179 infants with suspected CRS via the comprehensive approach, with 25 infants classified as laboratory-confirmed and 6 as clinically compatible. Surveillance sensitivity was highest for the simplified ophthalmic approach, while cataract-based surveillance had the highest proportion of confirmed cases. Conclusions: Simplified CRS surveillance, particularly focusing on detecting cataracts, can significantly contribute to monitoring the impact of rubella vaccine introduction. It could serve as an initial step towards comprehensive CRS surveillance, providing robust evidence to support rubella and CRS elimination efforts.

## 1. Introduction

Congenital rubella syndrome (CRS) results from a woman acquiring rubella infection during pregnancy, especially when the infection occurs during early pregnancy. As a result of rubella virus infection interrupting fetal development in utero, infants with CRS are born with one or more manifestations that may include congenital cataracts, glaucoma, hearing impairment, cardiac malformations, or developmental delay, each potentially causing life-long disabilities. The disability burden due to CRS is greatest in countries where the rubella vaccine has not yet been introduced. In 2010, the global burden of CRS was estimated at 105,000 CRS cases; in 2019, after 44 additional countries had introduced the rubella-containing vaccine (RCV), this burden decreased to 32,000 [1]. Further reductions in the global CRS burden can be achieved with the introduction of rubella vaccines into the remaining countries, reducing rubella transmission and the incidence of CRS.

By 2023, 175 countries had introduced the RCV. Five of the six World Health Organization (WHO) regions have established rubella and CRS elimination goals. The Eastern Mediterranean Region, of which Sudan is a member-state, is the only region yet to establish a rubella/CRS elimination goal. As of 2023, Sudan had not yet introduced RCV, which would be the first step towards rubella elimination.

Sudan has been collecting case-based rubella data through the measles surveillance system since 2006. The system has demonstrated endemic rubella, including outbreaks of rubella reported in 2014 in the Khartoum, Gezira, and Kasalla states [2]. In addition, previous studies have documented a large rubella immunity gap in Sudanese women of childbearing age in some areas [3,4]. The first study to confirm CRS cases collected clinical specimens in 2005 and 2006 from 31 infants suspected of having CRS from six hospitals in Khartoum. Among these, 11 were laboratory-confirmed with CRS, and rubella viral sequencing identified three genotypes of rubella virus (1E, 2B, and 1G) co-circulating in Sudan [5]. Subsequently, several studies have documented the occurrence of CRS in this country [3,6]. However, no CRS surveillance system has been established in Sudan.

Improving surveillance to better define the epidemiology of CRS in this country can improve the data available to assist decision-makers and program managers on how to prioritize RCV introduction, monitor the impact of its introduction, and, ultimately, assess progress to elimination. CRS surveillance is challenging to implement due to CRS’s broad spectrum of clinical manifestations, requiring the involvement of a wide range of clinics at surveillance sites to maximize sensitivity. These include general pediatric clinics and sub-specialty neurological, ophthalmology, otolaryngology, and cardiology clinics. Global standards for CRS surveillance [7] recommend the use of a sentinel site surveillance system to optimize the efficiency of case identification. With the challenges of implementing CRS surveillance, there is a pressing need for alternative approaches in low-resource settings, focusing on the optimal type of sentinel sites and the suspected case definition used to identify cases for notification.

A comprehensive retrospective study in Morocco suggested that congenital cataract surveillance was the most efficient case-finding strategy for CRS [8]. Of the common CRS manifestations, cataracts are among the most visible external manifestations and can be easily detectable by mothers or other community members. Infants with cataracts are, therefore, more likely to be presented for medical care early in life, which presents an opportunity for confirming CRS cases. However, reviews of prospective studies showed only a 16–25% incidence of congenital cataracts in infants with prospectively identified CRS [9,10]. The percentage of all childhood cataracts due to rubella varies depending upon the setting and rates of other infectious and noninfectious etiologies (e.g., genetic causes) [11].

Assessing alternative approaches to CRS surveillance, including simplified suspected case definitions, may improve surveillance quality by focusing on surveillance efforts. In the appropriate setting, a simplified case-based surveillance system where there is the ascertainment of infants with a focused set of clinical CRS manifestations rather than a broad range of manifestations may achieve the goals of CRS surveillance while maximizing sustainability in the country [4]. With cataracts being a possible manifestation that can increase the identification of CRS cases, a suspected case definition focused on congenital cataracts may be a useful approach to simplify the congenital rubella surveillance system. This is further facilitated by potential cataract surgery that often requires pre-operative evaluations that include a detailed cardiac exam. No analysis has been carried out to assess the feasibility and utility of a simplified CRS surveillance system to provide the data needed for decision-makers and program managers.

In Sudan, a CRS surveillance system with seven reporting sites was established, including general pediatrics hospitals, eye hospitals, and a cardiology hospital. We utilized this system to evaluate whether a focused CRS surveillance approach detecting a limited range of manifestations (e.g., ophthalmological or cardiac manifestations) or a focused clinical manifestation of CRS (cataracts) could be beneficial. We assessed this CRS surveillance approach according to the WHO CRS surveillance monitoring criteria and evaluated the performance of the system with three surveillance approaches: (1) simplified site surveillance for eye hospital-based surveillance (simplified-ophthalmic surveillance); (2) simplified site surveillance for heart hospital-based surveillance (simplified-cardiac surveillance); or (3) simplified-cataract-only surveillance for infants diagnosed with cataracts only (simplified-cataract surveillance).

## 2. Materials and Methods

### 2.1. CRS Surveillance System

Sites for the surveillance of CRS were established in seven hospitals in the Khartoum and Gezeira states. At each site, health care providers from relevant clinics were trained to detect and notify surveillance officers of suspected CRS cases for further investigation, with emphasis on the identification of cataracts. Relevant clinics included specialty clinics in cardiology, otolaryngology, ophthalmology, infectious diseases, pediatrics, and neonatology, though not all clinics existed at all sites. Sites themselves had wide, poorly defined catchment areas with cases presented from within two states (that contained 26% of the national population), though one site had referrals from throughout the country.

Infants who met the suspected case definition for CRS were enrolled between 15 February 2014 and 6 June 2017. The suspected case definition for CRS was any infant (less than 1 year of age) with any of the following: (1) ocular manifestations of CRS (e.g., cataract, pigmentary retinopathy or glaucoma), (2) congenital heart disease, (3) suspected hearing impairment, (4) suspected congenital TORCH infection (including toxoplasmosis or other infections (syphilis, varicella, parvovirus B19), rubella, cytomegalovirus, herpes virus), (5) purpura (another visible defect), or (6) clinician suspicion (or confirmation) of rubella infection in the mother during pregnancy.

After a surveillance officer was notified of a suspected CRS case, a case investigation form was completed, and appropriate specimens for rubella testing were collected. Case investigation forms collected data on maternal demographics, child demographics, pregnancy history, infant clinical history, and infant vaccination; clinical history included additional work-up for manifestations associated with CRS. Appropriate specimens included a serum specimen for rubella IgM and IgG testing and a throat swab and urine specimen for reverse-transcription–polymerase chain reaction (RT-PCR) testing. Ophthalmologists were requested to aspirate fluid from lenses removed during cataract surgery for rubella virus testing by RT-PCR whenever possible. Specimens were sent to the national laboratory for testing. Suspected case definition and final case classifications were adapted from the WHO case definitions (Table 1), with the final classification based upon laboratory results and a clinical case definition for cases with inadequate laboratory testing.

### 2.2. Data Analysis

The demographic data of the cases were described by site, sex, year of birth, age at presentation, place of delivery, and clinical presentation. Comparisons of factors between surveillance site and case classification were performed using the chi-square test; surveillance quality indicators for CRS [12] were calculated and compared across these subsets, and continuous variables were assessed by the Kruskal–Wallis test. These subsets evaluated the different approaches to surveillance, comparing comprehensive surveillance (all surveillance sites) with simplified surveillance approaches: simplified-site surveillance (i.e., simplified-ophthalmic surveillance and simplified-cardiac surveillance) and simplified-cataract surveillance by presenting manifestations. As sentinel sites do not have pre-defined catchment areas [13], the denominator used live births calculated based on national estimates for 2018 [14].

### 2.3. Trend Analysis

To assess the complementarity of surveillance for rubella by undertaking surveillance for CRS, we compared trends in rubella cases to trends of the detected CRS cases. Surveillance for rubella disease in Sudan was conducted through the measles surveillance system, which categorizes acute fever and rash cases as measles, rubella, or non-measles, and non-rubella (discarded) through the testing of sera detected in individuals with a fever, rash, and one or more of three symptoms (cough, coryza, conjunctivitis) using standard protocols (WHO, 2006). Because CRS is a result of rubella infection in a woman during early pregnancy, a well-functioning CRS system should detect trends in CRS that follow trends in rubella corresponding to the remaining gestation time from maternal infection to infant birth. We used cross-correlation to evaluate whether the CRS surveillance detected epidemiological trends that were reflective of the underlying rubella epidemiology in Sudan, specifically assessing the associated lag between rubella and CRS incidence in time series by describing the correlation accounting for the time delay from maternal infection to the onset of CRS (i.e., month of birth). This signal analysis is used to infer a relation between one time series and another; assuming that the specific rubella epidemiology among women of childbearing age is similar to the overall national trends, we would expect a positive cross-correlation between CRS cases and rubella cases, with a time lag corresponding to the remaining gestational time from maternal infection to the birth of the child. All statistical analyses were completed with R 3.2.2 (Vienna, Austria).

## 3. Results

The surveillance sites consisted of three general pediatrics hospitals, three eye hospitals, and one cardiology hospital, with sites including one or more different clinics or wards. Staff at all sites were trained and implemented the standard (comprehensive) protocol for CRS surveillance, with an emphasis on cataract manifestations.

### 3.1. Suspected Cases and Reporting

From 15 February 2014, to 6 June 2017, 179 eligible cases of suspected CRS were detected and notified, and 6 cases were notified beyond one year of age that were previously excluded. Of the total, 31 cases (17%) were reported by cardiology hospitals, 133 (74%) by eye hospitals, and 15 (8%) by pediatric hospitals (Table 2). A single eye hospital was responsible for 98 (55%) notifications.

Overall, infants meeting the suspected case definition were notified at a median age of 140 days (IQR: 86, 212). Age at reporting differed by site as the cardiology hospitals reported a median of 155 days of age (IQR: 87, 236), while pediatric hospitals reported a median of 9 days of age (IQR: 5, 16.5). Place of birth (home or health facility) varied significantly between sites (*p* = 0.0043), including home births being more common among those presenting to eye hospitals, followed by heart hospitals (53% vs. 27%, *p* = 0.0043) (Table 2).

Of the 179 suspected cases, 168 had at least one “Group A” clinical manifestation (Table 3) compatible with CRS, 136 (77%) had at least one eye finding, 47 (27%) had at least one heart finding, and one suspected case (1%) had hearing impairment, while eleven suspected cases did not have any Group A findings. Of the 136 cases with eye findings, 79 (44%) had cataracts, 57 (32%) had glaucoma, and 4 (10%) had pigmentary retinopathy. Among the 47 suspected CRS cases with cardiac manifestations, 30 cases (64%) had patent ductus arteriosus (PDA), 6 had ventricular septal defect (VSD) (13%), and 4 had peripheral pulmonary stenosis (PPS) (9%).

The final classification of the 179 cases was 25 laboratory-confirmed, 6 clinically compatible, and 148 discarded cases (Table 3). Of the seven suspected cases that died, three were lab-confirmed cases, and four were discarded cases. The age of reporting was similar between the final classifications, confirmed, and discarded cases (median 155 vs. 129 days, *p* = 0.07); reporting by sex, 53% were female (*p* = 0.13). None of the 57 suspected cases with glaucoma were laboratory-confirmed as CRS cases (two were clinically compatible), while all four pigmentary retinopathy cases were discarded. A clinical description of a subset of the cases is reported elsewhere [15].

### 3.2. System Performance

We assessed the performance of the different surveillance approaches using the standard surveillance indicators (Table 4), comparing comprehensive surveillance (all cases detected by the entire surveillance system), simplified-site surveillance from simplified-ophthalmic or simplified-cardiac site surveillance, or simplified-cataract surveillance. Comprehensive surveillance from all seven reporting sites had the highest sensitivity (1.32 per 10,000 live births nationwide), with surveillance from eye hospitals (0.98 per 104 live births) and surveillance for cataracts alone (0.58 suspected cases per 104 live births) having the highest sensitivity out of the simplified approaches. The system with the highest rate of case confirmation per suspected case was simplified-cataract surveillance with 24% (19 of 79 cases). Detecting cases early, within the first 3 months of life, was greatest with comprehensive surveillance (23%, 47 of 179 suspected cases). Serologic specimens were collected from 95% or more of cases at all surveillance approaches; simplified-cataract surveillance had the highest collection of virological specimens (61%).

### 3.3. Trend Analysis

**Figure 1:**
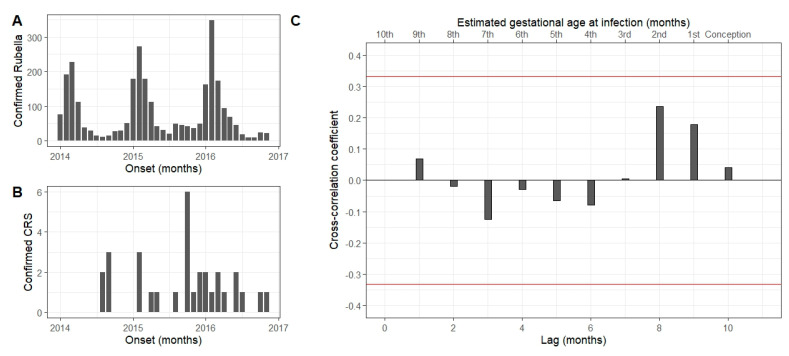
Comparison of the time trends of the rubella cases (panel **A**) and laboratory-confirmed congenital rubella syndrome (CRS) cases (panel **B**) in Sudan from January 2014 to December 2017. Panel **C** shows the cross-correlogram of the lag in CRS cases occurring following peaks in rubella cases. This figure indicates a positive (though not statistically significant, within the 95% confidence interval illustrated between the red lines) correlation with a lag of 8–10 months, corresponding to an increase in CRS cases following rubella cases occurring between conception and the third month of gestation.

Validating the complementarity of the trend of rubella cases detected through the measles surveillance system, the number of lab-confirmed rubella cases was compared to lab-confirmed CRS cases by month, and the relationship lags in the cross-correlation (Figure 1). Rubella cases are followed by equivalently elevated CRS cases peaking after 8 and 9 months (r = 0.23 and 0.18), which is lower than the 95% confidence band (0.331); observing CRS cases 4 months before rubella peaks, there is also a positive trend (r = 0.31) which could be associated with seasonal differences. This analysis suggests that the peak correlation between rubella and detected CRS cases occurs in CRS cases infected at 1–2 months gestation, though this was not statistically significant.

## 4. Discussion

Decreasing the burden of CRS is the primary purpose of rubella vaccine introduction and monitoring its introduction and impact requires adequate surveillance systems. The progress and achievement of rubella and CRS elimination requires high-quality surveillance to document changes. As expected, the comprehensive surveillance approach has the highest sensitivity, simplified CRS surveillance detection on a more focused and specific set of sites, or specific diagnoses are alternatives when resources are limited. One specific diagnosis that is an especially strong candidate is cataracts, which are one of the few visible CRS manifestations.

The simplified-cataract approach had a higher proportion of suspected cases confirmed as CRS (24%) than the other approaches. Cases identified from eye hospitals included cases with cataracts (*n =* 75), glaucoma (*n =* 55), and pigmentary retinopathy (*n =* 1); however, only cases with cataracts were laboratory-confirmed to be positive (*n =* 16) with two clinically compatible cases. However, two glaucoma cases were also clinically compatible. Notably, glaucoma cases presented at a younger age than cataract cases (median 112 vs. 178 days) and, therefore, are more likely to be positive for IgM testing. Both the simplified-ophthalmic and the simplified-cataract approaches had high rates of serological and virological sample collection but were less likely to detect confirmed CRS cases within the first 3 months of life compared to a simplified-cardiac approach.

While this assessment may reflect the proof of concept of simplified surveillance, these results also reflect the quality of the sites selected. Optimal sentinel site surveillance, including CRS, is driven by careful site selection based on efficiency, patient flow, and other factors. For example, simplified-ophthalmic surveillance performed compared well to a comprehensive surveillance approach involving multiple manifestations of the case definition and various types of surveillance sites. Noting that the simplified-ophthalmic approach was driven by one site with a nationwide referral network, this highlights the value of including surveillance sites with a large catchment area for CRS surveillance. Some selection factors are learned through experience, such as the proportion of cases that are laboratory-confirmed, which varied between similar sites (e.g., between eye hospitals).

Surveillance for CRS may complement rubella surveillance, with temporal trends in CRS being associated with rubella disease. Cross-correlation analysis identified a weak, positive, but not statistically significant correlation between national rubella epidemiology and CRS epidemiology, peaking at around 8 months of lag, which may suggest that the CRS system captures the same epidemiological phenomena as rubella case-based surveillance and, thus, serves as a useful complement to case-based surveillance. Cataracts are most often associated with infection during the second or third month of gestation [16], and the cross-correlation results were consistent with this finding.

CRS surveillance does monitor trends and complements surveillance for rubella, as neither comprehensive nor simplified surveillance approaches capture the total burden of CRS in Sudan, as the true incidence is likely higher. This underestimation of incidence can occur for several reasons, including estimating the catchment area and case ascertainment. The catchment area of all but one of the sites was limited to two of eighteen states (26% of the national population); one eye site had a national catchment. Case ascertainment is not consistent with the expected proportions of CRS-affected infants. For example, hearing impairment is the most common manifestation, seen in approximately 57% of confirmed cases [10] but underreported and identified in only one suspected case. Cataracts are overreported and are expected to be reported in 16–25% of cases [9,10], though they were seen in 64% of confirmed cases from the comprehensive surveillance approach.

The CRS surveillance system in Sudan has similarities and differences to data from other systems prior to the rubella vaccine introduction [17,18,19,20,21,22]. The CRS incidence for simplified-cataract surveillance (0.58 cases/10,000 live births) and simplified-ophthalmic surveillance (0.98 cases/10,000 live births) in Sudan was similar to 1.0 [18] but lower than two other systems, 3.9, 4.0 [17,22], noting how catchment areas differed to the point that several systems did not attempt to calculate incidence [19,20]. The percentage of suspected cases that were laboratory-confirmed varied between systems, and the system in Sudan (17%) is similar to most systems at 18–22% [17,18,22] but lower than the high of 34% [19]. As expected, the proportion of suspected cases with cataracts from Sudan (44%) was greater than other systems, which ranged from 22% [21] to 37% [19]; concomitantly, a lower percentage of suspected cases with cardiac findings (27%) were reported from Sudan than the other systems which ranged from 46% [17] to 65% [19]. In summary, simplified surveillance (especially cataract and ophthalmic approaches) from Sudan has a greater percentage of cases with cataracts and a lower percentage of positive cases while having similar or lower incidence rates.

This evaluation has several limitations. Clinical case data were limited to a few health facilities in Sudan, which may not reflect the heterogeneity of the disease in Sudan. Surveillance was unable to be continued with high quality beyond 2017 due to funding, staff turnover, and other operational challenges, highlighting the reality of maintaining surveillance systems in low–middle income countries. The analysis focuses on detection but does not account for the human and financial resource burdens of the different surveillance approaches. Most cases were identified through eye hospitals, which had the lowest efficiency rate, highlighting the importance of assessing resources in future evaluations to strike a balance between sensitivity and specificity in system development. The overall background rate of congenital cataracts and their various causes varies globally [23]; this limits between-country incidence, making cataract-based surveillance approaches less comparable between countries. In addition, unique situations in this surveillance system (e.g., an eye hospital with a nationwide catchment area) may limit generalizability. Lastly, the surveillance training had an increased emphasis on the detection of cataracts relative to other symptoms, which may have increased the detection of cataracts compared to other CRS manifestations.

## 5. Conclusions

A comprehensive CRS surveillance system is the standard for monitoring elimination, as defined in the WHO Surveillance Standards [7], complementing surveillance for rubella. A simplified approach may be an alternative where there are limited resources, especially during the rubella vaccine introduction period or when initially establishing a CRS surveillance network. A simplified system can achieve some key goals of CRS surveillance, including documenting the impact of vaccine introduction and identifying infants with CRS to institute control measures and facilitate the early provision of appropriate medical care for the infant. We assessed simplified CRS surveillance within the specific context of Sudan’s health care infrastructure and epidemiology. A simplified surveillance system built primarily on eye hospitals or eye findings (either through limiting the scope to eye hospitals and clinics or through a simplified cataract definition for suspected cases) would be effective in detecting CRS cases and may achieve the CRS surveillance goals of this country while reducing the operational burden and improving sustainability. Cases identified by such a system may have different clinical profiles than other CRS surveillance systems. Sudan has a well-developed network of clinical sites for ophthalmological screening and referral for treatment due to a relatively high baseline prevalence of congenital and childhood cataracts [24], making a simplified-ophthalmic approach more feasible than a simplified-cataract approach, which can then be expanded to increase the sensitivity of surveillance for CRS.

While it is rubella vaccines that will decrease the burden of CRS, we conclude that simplified surveillance can play an important role in monitoring the impact of rubella vaccine introduction, while a more sensitive surveillance approach would provide more robust evidence for the elimination of rubella and CRS.

## Figures and Tables

**Table 1:** Definitions and criteria used to classify congenital rubella syndrome (CRS) cases.

Final Case Classification	Definition
Laboratory-confirmed CRS	A suspected case ^1^ with one or more Group A symptoms and laboratory confirmation.
Clinically compatible CRS	A suspected case ^1^ with either (1) two or more Group A symptoms, or (2) one Group A and one or more Group B symptoms and inadequate laboratory testing.
Discarded case	A suspected case ^1^ with adequate laboratory testing and no laboratory confirmation, or a case that does not meet the clinically compatible case definition.
Congenital rubella infection	A suspected case ^1^ with no Group A symptoms and laboratory confirmation.
Group A symptoms: congenital cataracts, congenital glaucoma, congenital heart disease, hearing impairment, or pigmentary retinopathy. Group B symptoms: purpura, splenomegaly, microcephaly, developmental delay, meningoencephalitis, radiolucent bone disease, and jaundice that begins within 24 h after birth.	
Adequate laboratory testing: a single IgM specimen result for <6 months of age, or two sequential IgG test results (at least 2 weeks apart) for children 6 m–<12 m of age. Laboratory confirmation: occurs when an infant has (1) a positive rubella IgM, (2) positive RT-PCR test <12 months of age (no vaccination history), and/or (3) two sequential specimens with stable or increasing IgG levels.	

^1^ The suspected case definition used to ascertain cases was an infant with one or more of the following conditions: cataracts, glaucoma, pigmentary retinopathy, suspected hearing impairment, congenital heart disease, purpura, suspected congenital infection, and suspected or confirmed rubella infection of the mother during pregnancy.

**Table 2:** Demographics of suspected cases detected by type of site, including heart, eye, or pediatric hospitals. Associated *p*-values use the Chi-square test of independence for proportions between heart, eye, and pediatric hospitals and the Kruskal–Wallis test for medians.

	Total/ Comprehensive *n =* 179	Heart Hospitals *n =* 31	Eye Hospitals *n =* 133	Pediatric Hospitals *n =* 15	Test Statistic
Age at notification, median days (IQR)	140 (86, 212)	155 (87, 236)	154 (96, 214)	9 (5, 16.5)	*p* < 0.001
Male Sex, *n* (%)	94 (53%)	14 (45%)	70 (53%)	10 (67%)	*p* = 0.391
Place of delivery, *n* (%)					
Health facility	90 (51%)	20 (67%)	59 (45%)	11 (73%)	*p* = 0.0043
Home	81 (46%)	8 (27%)	70 (53%)	3 (20%)	
Not reported	5 (3%)	2 (7%)	2 (2%)	1 (7%)	
Eye findings, *n* (%)	136 (77%)	7 (23%)	127 (97%)	2 (13%)	*p* < 0.0001
Hearing impairment, *n* (%)	1 (1%)	0 (0%)	0 (0%)	1 (7%)	*p* < 0.085
Congenital heart disease, *n* (%)	47 (27%)	30 (97%)	11 (8%)	6 (40%)	*p* < 0.001

**Table 3:** Demographics of cases by final classification, subdividing the total confirmed cases by those that were confirmed with laboratory results and those that were clinically compatible.

	Suspected *n =* 179	All Confirmed *n =* 31	Lab-Confirmed *n =* 25	Clinically Compatible *n =* 6	Discarded *n =* 148
Days of age at reporting Median (IQR)	140 (87–216)	154 (88–234)	145 (64–218)	233 (174–263)	129 (86–198)
Male sex (*n*, %)	94 (53%)	12 (39%)	11 (44%)	1 (17%)	82 (55%)
Eye findings (*n*, %)	136 (77%)	24 (77%)	19 (76%)	5 (83%)	102 (77%)
Cataracts	79 (44%)	22 (71%)	19 (76%)	3 (50%)	58 (38%)
Glaucoma	57 (32%)	2 (6%)	0 (0%)	2 (33%)	55 (36%)
Pigmentary retinopathy	4 (2%)	0 (0%)	0 (0%)	0 (0%)	4 (2%)
Hearing Impairment (*n*, %)	1 (1%)	0 (0%)	0 (0%)	0 (0%)	1 (1%)
Heart findings	47 (27%)	19 (61%)	13 (52%)	6 (100%)	28 (19%)
Patent ductus arteriosus	30 (17%)	14 (45%)	10 (40%)	4 (67%)	16 (11%)
Primary pulmonary stenosis	4 (2%)	1 (3%)	1 (4%)	0 (0%)	3 (2%)
Ventral septal defect	6 (3%)	1 (3%)	1 (4%)	0 (0%)	5 (3%)
Other defect	2 (1%)	0 (0%)	0 (0%)	0 (0%)	2 (1%)
Clinical criteria reported					
2 “A”	18 (10%)	13 (43%)	8(32%)	5 (83%)	5 (3%)
1 “A” and ≥1 “B”	10 (6%)	3 (10%)	2 (8%)	1 (17%)	7 (5%)
1 “A” and 0 “B”	137 (78%)	13 (43%)	13 (54%)	0 (0%)	124 (85%)
None of the above	11 (6%)	1 (3%)	1 (4%)	0 (0%)	10 (7%)
Reporting site					
Cardiology hospital	31 (17%)	8 (26%)	5 (20%)	3 (50%)	23 (16%)
Eye hospital	133 (74%)	19 (61%)	16 (64%)	3 (50%)	114 (77%)
Pediatric hospital	15 (8%)	4 (13%)	4 (16%)	0 (0%)	11 (7%)

**Table 4:** Surveillance for congenital rubella syndrome (CRS) indicators comparing the indicators based upon the scope of the surveillance system implemented, from comprehensive assessments to surveillance simplified by site and simplified for cataracts as a major case definition. For each indicator, the box with the most favorable indicator is colored green, and the least favorable is colored gray.

Type of CRS Surveillance	Comprehensive	Simplified Site		Simplified Manifestation
Indicator	All cases (*n =* 179)	Simplified-cardiac surveillance (*n =* 31)	Simplified- ophthalmic surveillance (*n =* 132)	Simplified-cataract surveillance (*n =* 79)
Number of reporting sites/sentinel sites	7	1	3	5
Sensitivity—national annual rate of suspected CRS cases. Target ≥ 1 per 10,000 live births	1.32	0.23	0.98	0.58
Case confirmation rate (% efficiency, confirmed cases)	14% (*n =* 25)	16% (*n =* 5)	12% (*n =* 16)	24% (*n =* 19)
Confirmed CRS cases detected within 3 months of birth (%, confirmed cases)	23% (*n =* 47)	23% (*n =* 8)	16% (*n =* 25)	13% (*n =* 14)
Suspected case with adequate blood specimens for serology testing (%, cases)	95% (*n =* 170)	87% (*n =* 27)	96% (*n =* 128)	95% (*n =* 75)
Proportion of serological samples from suspected cases received within 5 days of collection	95% (*n =* 157)	96% (*n =* 27)	95% (*n =* 116)	96% (*n =* 67)
The proportion of suspected cases with an adequate virological specimen	44% (*n =* 79)	45% (*n =* 14)	42% (*n =* 56)	61% (*n =* 48)

## Data Availability

The data presented in this study are available on request from the first author due to the data being government data.
